# Fluvastatin in the first-line therapy of acute coronary syndrome: results of the multicenter, randomized, double-blind, placebo-controlled trial (the FACS-trial)

**DOI:** 10.1186/1745-6215-11-61

**Published:** 2010-05-25

**Authors:** Petr Ostadal, David Alan, Jiri Vejvoda, Jiri Kukacka, Milan Macek, Petr Hajek, Martin Mates, Milan Kvapil, Jiri Kettner, Martin Wiendl, Ondrej Aschermann, Josef Slaby, Frantisek Holm, Peter Telekes, David Horak, Peter Blasko, David Zemanek, Josef Veselka, Jana Cepova

**Affiliations:** 1 Heart Center, Department of Cardiology, Na Homolce Hospital, Prague, Czech Republic; 2 Department of Cardiology, University Hospital Motol, Prague, Czech Republic; 3 Department of Clinical Biochemistry and Pathobiochemistry, University Hospital Motol and Charles University, 2nd Faculty of Medicine, Prague, Czech Republic; 4 Institute of Biology and Medical Genetics, University Hospital Motol and Charles University, 2nd Faculty of Medicine, Prague, Czech Republic; 5 Department of Internal Medicine, University Hospital Motol and Charles University, 2nd Faculty of Medicine, Prague, Czech Republic; 6 Department of Cardiology, Institute for Clinical and Experimental Medicine, Prague, Czech Republic; 7 Department of Medicine, Hospital Kolin, Kolin, Czech Republic; 8 Department of Cardiology, Regional Hospital Liberec, Liberec, Czech Republic; 9 Internal Medicine Department, Faculty Hospital Nitra, Nitra, Slovakia

## Abstract

**Background:**

Statins have been proved to be effective in reduction of mortality and morbidity when started in the early secondary prevention in stabilized patients after acute coronary syndrome (ACS). The safety and efficacy of statin administration directly in the first-line therapy in unstable ACS patients is not clear. The aim of our study was, therefore, to assess the effect of statin treatment initiated immediately at hospital admission of patients with ACS.

**Methods:**

The trial was stopped prematurely after enrollment of one hundred and fifty-six patients with ACS that were randomized at admission to fluvastatin 80 mg (N = 78) or placebo (N = 78). Study medication was administered immediately after randomization and then once daily for 30 days; all patients were then encouraged to continue in open-label statin therapy and at the end of one-year follow-up 75% in the fluvastatin group and 78% in the placebo group were on statin therapy.

**Results:**

We did not demonstrate any difference between groups in the level of C-reactive protein, interleukin 6, and pregnancy-associated plasma protein A on Day 2 and Day 30 (primary endpoint). Fluvastatin-therapy, however, significantly reduced one-year occurrence of major adverse cardiovascular events (11.5% vs. 24.4%, odds ratio (OR) 0.40, 95% CI 0.17-0.95, P = 0.038). This difference was caused mainly by reduction of recurrent symptomatic ischemia (7.7% vs. 20.5%, OR 0.32, 95% CI 0.12-0.88, P = 0.037).

**Conclusions:**

This study failed to prove the effect of fluvastatin given as first-line therapy of ACS on serum markers of inflammation and plaque instability. Fluvastatin therapy was, however, safe and it may reduce cardiovascular event rate that supports immediate use of a statin in patients admitted for ACS.

**Trial registration:**

NCT00171275

## Background

Statins (3-hydroxy-3-methylglutaryl coenzyme A reductase inhibitors) are cholesterol-lowering drugs, very effective in the reduction of mortality and non-fatal cardiovascular events rates in both primary and secondary prevention of ischemic heart disease. It was, however, found that statin administration results not only in the reduction of total- and LDL-cholesterol (statin-induced blockade of cholesterol-synthesis) but also in a number of cholesterol-independent effects, known as "pleiotropic" effects of statins. Development of acute coronary syndrome (ACS) involves several pathogenic pathways that can be inhibited by statins, including cholesterol accumulation in plaque, endothelial dysfunction, activation of inflammation and thrombus formation; this fact supports the idea to use statins also under conditions of ACS. Recently, several large prospective, controlled clinical trials have been published, showing safety and efficacy of statins, when administered in stabilized patients early after ACS [1-3]. An increasing number of observations demonstrates, however, that statins may play a beneficial role not only in early secondary prevention but also directly in the therapy of ACS, i.e. when statin treatment is started as first-line care in clinically unstable patients. This therapeutic approach is supported by (i) experimental studies, showing the protective effect of statins under the condition of acute ischemia [4-8], (ii) analysis of different registers and trials, demonstrating better prognosis of statin-treated patients with ACS [9-11], and (iii) small clinical trials, describing an improvement in some pathogenic factors and markers as a result of immediate statin therapy [12-18]. The well-controlled prospective trial showing the efficacy of statin administration in first-line therapy of ACS is, however, still lacking. We have designed, therefore, a prospective, placebo-controlled trial, testing the hypothesis that statin, when administered at admission in patients with ACS, suppresses the inflammatory burden and improves clinical outcomes.

## Methods

### Overview

The study design has been published previously [19]. Briefly, the FACS trial is a prospective, multicenter, randomized, double-blind, placebo-controlled study in patients with ACS. Patients were enrolled between November 2003 and February 2006 from 5 sites in the Czech Republic, follow-up was completed in March 2007. The study complied with the Declaration of Helsinki, protocol and informed consent form were reviewed and approved by the multicenter and institutional ethics committees before study initiation. Eligible patients were randomized to one of two treatment groups immediately after hospital admission (within one hour). One group was assigned 80 mg fluvastatin XL once daily, the other was assigned placebo. The co-primary endoints were the levels of CRP, IL-6, and PAPP-A/proMBP at days 2 and 30. The combined secondary endpoint was the one-year occurrence of a major adverse cardiovascular events (MACE), defined as death, nonfatal myocardial infarction (MI), recurrent symptomatic myocardial ischemia, urgent revascularization.

### Study population

Patients were eligible for the enrolment if they had ACS with ST-elevation (rest chest pain less than 12 hours before admission and ≥ 1 mm ST-segment elevation in 2 or more contiguous leads or new left bundle branch block on ECG) or non-ST elevation ACS (rest chest pain during the past 48 hours and ≥ 1 mm ST segment depression or negative T waves in 2 or more contiguous leads). Exclusion criteria were: concomitant active liver disease or persistent elevation of transaminases more than three times above the upper limit of normal, history of lipid lowering therapy less than 30 days before index event, known allergy for fluvastatin or any present additives in the drug, disability of oral drug administration, disability of follow-up, pregnancy or nursing, women of fertile age without effective contraception, suspicions of muscle disease like myositis, subjects younger than 18 years, creatine kinase ≥ 5 times of the upper limit of normal range with other casual explanation than presence of myocardial infarction.

### Study design

Following signed informed consent, blood samples were taken for examination of serum markers of inflammation and plaque instability (CRP, IL-6, and PAPP-A/proMBP) and patients were randomized to fluvastatin 80 mg or placebo immediately p.o. (within one hour after hospital admission). Medical history and physical examination, standard 12-lead ECG, blood lipid profile, and liver function study were performed as part of routine admission procedure. Fluvastatin 80 mg/placebo had then been administered once daily for 30 days. Follow-up examination of inflammatory markers (CRP, IL-6, and PAPP-A/proMBP) was performed on day 2 and day 30. Follow-up visits were scheduled: pre-discharge, and day 30, 90, 180, 360. Blood liver function and creatine kinase tests were done pre-discharge and at 30-day visits. At the 30-day visit the lipid profile was also examined and study medication was withdrawn. All visits included assessment of the history of cardiovascular events, concomitant medication and other relevant medical history.

During follow-up, no specific recommendations were made with respect to diagnostic and therapeutic strategy, except that other lipid-lowering drugs should be omitted since randomization until day 30. All management decisions were left to the discretion of each patient's treating physician.

### Laboratory analysis

Serum was prepared immediately after blood sampling and stored frozen at -70°C until laboratory assessment. IL-6 in serum was measured by a solid phase, enzyme-labeled, chemiluminescent sequential immunometric assay IMMULITE 1000 IL-6 by IMMULITE 1000 immunochemical system (DPC, USA). For the quantitative determination of C-reactive protein in serum was used a turbidimetric immunoassay Wako CRP-HS (Wako Chemicals, Germany); the analysis was performed by ADVIA 1650 biochemical analyzator (Siemens, USA). The levels of PAPP-A/proMBP were determined by Kryptor system (Brahms, Germany).

### Statistical analysis

Baseline characteristics of the fluvastatin group and placebo group were compared using *t*-test, Χ^2 ^test or Fisher test. Repeated measures ANOVA with three within subject levels (Day 0, 2, and 30) were used to test the differences of IL-6, CRP, and PAPP-A between the fluvastatin group and placebo group. Logarithmic transformation of continuous data prior to the analysis was used to achieve normally distributed data and homogeneity of variances in subgroups. The incidence of cardiovascular events in the active treatment group was compared with the incidence in the placebo group with an Χ^2 ^test or Fisher test. For time-to-event analysis a log-rank test was used. Survival functions were estimated according to the Kaplan-Meier product-limit method. All the comparisons were done on intention-to-treat basis and *P *< .05 was considered statistically significant.

### Safety

The principal safety concerns were hepatic dysfunction and myopathy. If a patient's serum transaminase levels were persistently elevated to > 3 times above the upper limit of normal, the study medication should be discontinued per protocol. Similarly, the study medication should be stopped if the patient developed muscle pain, weakness, or tenderness in association with a serum creatine kinase level >10 times above the upper limit of normal.

### Sample size

The trial was originally planned with 1,000 patients to ensure adequate power to detect significant treatment benefit of 80 mg fluvastatin with respect to 30-day decrease of CRP and IL-6 (primary endpoint) and combined secondary endpoint. With 500 patients randomized to 80 mg fluvastatin and 500 patients randomized to placebo the trial had a more than 80% power to detect a decrease in CRP level by 1.36 mg/L and a decrease in IL-6 level by 1.09 ng/L; calculations were based on a two-sample *t*-test. Estimated combined secondary endpoint rate was 20%. Based on comparison of proportions with *p *= 0.05 test significance, the trial had more than 80% power to detect a decrease by 33% in the combined secondary endpoint.

## Results

**Figure 1:**
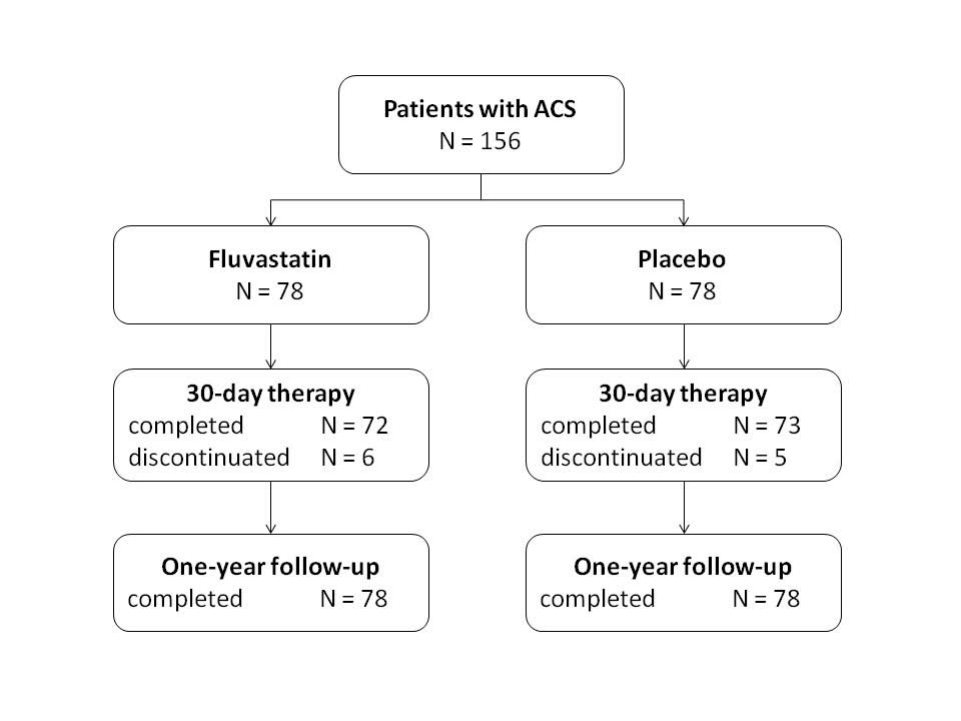
**Flow chart of subjects included in the trial**. Number of patients screened was not determined. All patients completed one-year follow-up. ACS, acute coronary syndrome.

The study was stopped prematurely due to slow recruitment rate. A total of one-hundred and fifty-six patients were randomized and all completed the follow-up; seventy-eight subjects were assigned to each group (Figure 1). We did not capture any unexplained or treatment-related impaired liver function or myopathy. Fluvastatin and placebo groups were comparable in the baseline characteristics including medical history, clinical characteristics, and concomitant medication (Table 1). At day 30, open-label statin therapy was initiated in all patients; at the end of one-year follow-up, 75% of patients were treated with statin in the fluvastatin group and 78% in placebo group (ns.). We did not find any difference between groups also in the use of acetylsalicylic acid (84% vs. 87%), ticlopidin or clopidogrel (16% vs. 20%), ACE inhibitors (77% vs. 72%), and beta-blockers (84% vs. 81%).

**Table 1:** Group characteristics.

	Fluvastatin N = 78	Placebo N = 78	*P*
**Demographical data**			
Age (years; mean ± SD)	60.9 ± 11.5	63.2 ± 11.3	ns.
Gender			ns.
Male	70.5	65.4	
Female	29.5	34.6	
Weight (kg; mean ± SD)	84.1 ± 13.2	82.7 ± 14.9	ns.
Smoking status			ns.
Current smoker	42.3	50.0	
Non-smoker	43.6	39.7	
Former smoker	14.1	10.3	
			
**History**			
IHD	16.7	25.6	ns.
MI	5.1	10.3	ns.
PCI	1.3	5.1	ns.
CABG	0	1.3	ns.
Diabetes	17.9	20.5	ns.
Hypercholesterolemia	11.5	11.5	ns.
Hypertension	51.3	51.3	ns.
Stroke	3.8	2.6	ns.
PVD	2.6	2.6	ns.
			
**Clinical data**			
Heart rate (bpm; mean ± SD)	76.1 ± 18.1	72.2 ± 16.5	ns.
SBP (mmHg; mean ± SD)	138.4 ± 29.2	141.1 ± 25.2	ns.
DBP (mmHg; mean ± SD)	81.9 ± 17.4	80.8 ± 18.0	ns.
Acute coronary syndrome type			ns.
STE ACS	60.3	69.2	
Non-STE ACS	39.7	30.8	
Duration of symptoms (Hrs; mean ± SD)	10.1 ± 8.9	8.0 ± 5.9	ns.
Killip class			ns.
Killip I	88.5	87.2	
Killip II	9.0	11.5	
Killip III	1.3	0	
Killip IV	1.3	1.3	
Total cholesterol at admission (mmol/L; mean ± SD)	5.5 ± 1.3	5.4 ± 1.1	ns.
Troponin I at admission (μg/L; mean ± SD)	14.7 ± 29.1	8.3 ± 14.1	ns.
Peak troponin I (μg/L; mean ± SD)	78.3 ± 110.8	78.7 ± 93.9	ns.
LV ejection fraction (mean ± SD)	48.0 ± 11.4	49.0 ± 10.9	ns.
			
**Therapy**			
Coronary angiography	98.7	98.7	ns.
Coronary intervention	87.2	91.0	ns.
PCI	75.6	87.2	0.098
CABG	11.5	3.8	0.130
ASA	98.7	98.7	ns.
IIb/IIIa inhibitors	5.1	5.1	ns.
UFH/LMWH	82.1	84.6	ns.
Beta-blockers	88.5	88.5	ns.
Diuretics	6.4	16.7	0.077
Clopidogrel	71.8	84.6	0.080
ACE inhibitors	66.7	69.2	ns.
Vyšš í odborná škola	53	52	+1
			

Values are expressed in per cent or as indicated. IHD, ischemic heart disease; MI, myocardial infarction; PCI, percutaneous coronary intervention; CABG, coronary artery bypass surgery; PVD, peripheral vascular disease; SBP, systolic blood pressure; DBP, diastolic blood pressure; STE, ST-segment elevation on ECG; ACS, acute coronary syndrome; LV, left ventricle; PCI, percutaneous coronary intervention; CABG, coronary artery bypass surgery; ASA, acetylsalicylic acid; UFH, unfractionated heparin; LMWH, low-molecular-weight heparin; ACE, angiotensin-converting enzyme.

**Figure 2:**
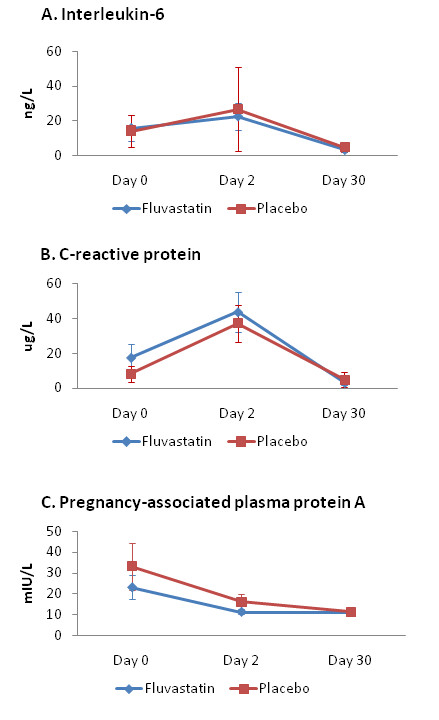
**Levels of interleukin 6, CRP, and PAPP-A**. D0, day 0; D2, day 2; D30, day 30. Panel A: serum levels of interleukin 6. Panel B: serum levels of C-reactive protein (CRP). Panel C: serum levels of pregnancy-associated plasma protein A (PAPP-A). No significant differences between Fluvastatin group and Placebo group were detected.

**Figure 3:**
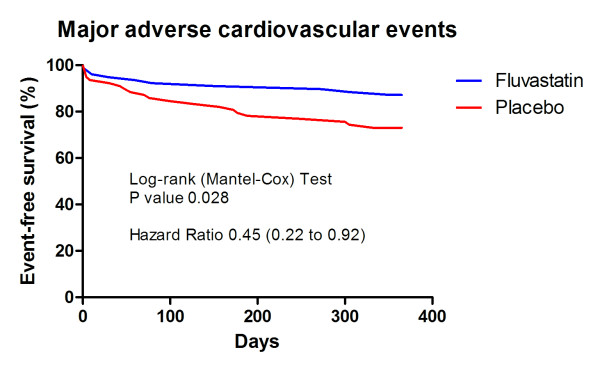
**Event-free survival of major adverse cardiovascular events**. Major adverse cardiovascular events (MACE) were defined as death, nonfatal myocardial infarction, symptomatic recurrent ischemia, or urgent revascularization.

We were unable to find significant differences between the groups in the levels of CRP, IL-6, and PAPP-A (primary endpoints) (Figure 2). We have observed, however, significantly lower occurrence of MACE and significantly longer event-free survival in the fluvastatin group (Table 2, Figure 3). Although the absolute numbers of all single monitored clinical events were lower in the fluvastatin group, it reached statistical significance only for recurrent symptomatic myocardial ischemia and number of new hospitalizations (Table 2). At 30 days comparing to placebo group, fluvastatin decreased the level of total cholesterol by 24.5% (4.40 ± 0.96 vs. 5.75 ± 1.26 mmol/L, p < 0.0001) LDL-cholesterol by 29.5% (2.75 ± 0.8 vs. 3.89 ± 0.97 mmol/L, p < 0.0001) whereas levels of HDL-cholesterol (1.14 ± 0.32 vs. 1.24 ± 0.32 mmol/L, ns.) and triglycerides (2.18 ± 0.95 vs. 2.09 ± 0.98 mmol/L, ns) remained unchanged. We did not observe any difference in creatine kinase (1.58 ± 0.85 vs. 1.44 ± 0.87 μkat/L, ns.) and liver enzymes (aspartate aminotransferase: 0.47 ± 0.26 vs. 0.44 ± 0.14 μkat/L, ns.; alanine aminotransferase: 0.52 ± 0.24 vs. 0.52 ± 0.27 μkat/L, ns.), all values at 30 days were lower than 3× ULN.

**Table 2:** One-year occurrence of major adverse cardiovascular events.

	Fluvastatin N = 78	Placebo N = 78	OR	95%CI	*P*
Death	1 (1.3)	4 (5.1)	0.24	0.02-2.20	0.37
Nonfatal MI	2 (2.6)	4 (5.1)	0.49	0.08-2.74	0.68
UAP	6 (7.7)	16 (20.5)	0.32	0.11-0.87	0.037
Urgent revascularization	6 (7.7)	14 (17.9)	0.38	0.14-1.05	0.09
PCI	5 (6.4)	13 (16.7)	0.34	0.12-1.01	0.08
CABG	1 (1.3)	2 (2.6)	0.49	0.04-5.56	1.0
Stroke	1 (1.3)	3 (3.8)	0.32	0.03-3.19	0.62
New hospitalization	14 (17.9)	34 (43.6)	0.28	0.13-0.59	<0.001
Death+MI	3 (3.8)	8 (10.3)	0.35	0.09-1.37	0.21
Death+MI+UAP++Urgent revascularization	9 (11.5)	19 (24.4)	0.40	0.17-0.95	0.038
Death+MI+UAP+Stroke++Urgent revascularization	10 (12.8)	21 (26.9)	0.40	0.17-0.92	0.044
					

Values are expressed as No. (%). MI, myocardial infarction; UAP, unstable angina pectoris; PCI, percutaneous coronary intervention; CABG, coronary artery bypass surgery

## Discussion

The major observation of the present trial is the reduction of cardiovascular events after administration of fluvastatin in first-line therapy of ACS patients treated with primary PCI or early invasive strategy and according to the current recommendations despite inability to find any difference in CRP, IL-6, and PAPP-A levels. The FACS trial was, however, stopped prematurely and the final number of patients enrolled is much lower than was expected. The explanation of the low recruitment rate can be only speculative: during the enrolment period the results of several large clinical trials with initiation of statin therapy early after ACS were published leading to unwillingness of investigators to delay the statin administration for 30 days in the placebo group; furthermore, many physicians initiate statin therapy intuitively earlier in the less stable ACS patients, still despite insufficient evidence.

We were unable to find even a trend to reduction of CRP and IL-6 levels by fluvastatin. This observation is in contradiction with several other studies showing decrease in the various inflammatory markers with early statin therapy in patients with non-ST-elevation ACS [13-16,20,21]. This discrepancy can be at least partly explained by different types and doses of statin used and also by the recruitment of patients with both types of ACS in FACS trial leading to increased scattering of infarct size and pro-inflammatory triggers. Cholesterol-lowering effect of fluvastatin was, however, fully expressed and based on the current view on the signaling pathways participating in the "pleiotropic effects of statins" it can be supposed that also anti-inflammatory pathways were activated but probably not intensively enough to outweigh the strong pro-inflammatory milieu. Our observation that PAPP-A levels were not influenced by fluvastatin therapy is in agreement with the literary data, showing no [22] or only mild [23] effect of statins on PAPP-A.

We have found statistically significant, approximately 50% reduction of the major cardiovascular events - the combined secondary endpoint. This unexpected considerable effect of fluvastatin was apparent across all the monitored single events including death, non-fatal myocardial infarction, recurrent ischemia, urgent revascularization, or stroke; statistical significance was, however, reached just in the occurrence of recurrent ischemia and combined endpoints. There was also significantly better event-free survival in the fluvastatin group. Moreover, fluvastatin-treated patients experienced less than half of new hospitalizations occurring in the placebo group. The FACS trial had, however, insufficient power to detect a difference in clinical outcomes and these observations may be influenced by the low number of participants. Similar reduction rate after fluvastatin therapy as we have shown for MACE was also recently reported for cardiovascular mortality or myocardial ischemia in the DECREASE III trial [24]. It can only be speculated that the beneficial effect of fluvastatin in the FACS trial was not driven by its anti-inflammatory properties, or was driven by anti-inflammatory properties not indicated by the measured biomarkers.

The FACS trial was designed and initiated before the publication of the large, well-controlled, prospective trials with statin therapy started in stabilized patients earlier after ACS. From the current point of view, the 30-days delay in statin administration after ACS in the FACS trial placebo-arm seems to be very long compared to the maximum of ten days in PROVE IT-TIMI 22 trial [2], five days in A to Z trial [3], or even four days in the MIRACL study [1] that enrolled, however, only conservatively treated patients. We cannot, therefore, answer the question whether there is incremental benefit from the immediate initiation of statin in the first-line therapy over its postponement for four to ten days. The FACS trial has shown that therapeutic strategy with administration of fluvastatin at admission in unstable ACS patients might be superior to the initiation of statins in stabilized patients 30 days after admission.

## Conclusions

Up to the best knowledge the FACS trial (Fluvastatin in the therapy of Acute Coronary Syndrome) is the first randomized, double blind, placebo-controlled study focused on the effects of statin administration in the first-line therapy of acute coronary syndrome. We have shown that in ACS patients treated with early invasive strategy and according to the current recommendations for pharmacological interventions, fluvastatin therapy started at admission does not influence serum markers of inflammation and plaque instability, however, it is safe and may improve clinical outcomes in these patients.

## Competing interests

The FACS trial was supported by the Czech Ministry of Health and by Novartis Pharma Czech republic. Moreover, authors received research funds, consultancy honoraria, and speakers honoraria from AstraZeneca, Hoffman La Roche, Novartis, Pfizer, and Servier.

## Authors' contributions

PO, DA, JiV, MaM, MK, MiM, and PB: conception and design of the trial. JKu, MiM, and JC: laboratory data analysis and interpretation. PO, DA, JiV, PH, JKe, MW, OA, JS, FH, PT, DH, DZ: clinical data analysis and interpretation. PO, DA, JiV: manusript drafting. MiM, JoV: critical revision of the manuscript. All authors read and approved the final manuscript.
